# ROMOP: a light-weight R package for interfacing with OMOP-formatted electronic health record data

**DOI:** 10.1093/jamiaopen/ooy059

**Published:** 2019-01-04

**Authors:** Benjamin S Glicksberg, Boris Oskotsky, Nicholas Giangreco, Phyllis M Thangaraj, Vivek Rudrapatna, Debajyoti Datta, Remi Frazier, Nelson Lee, Rick Larsen, Nicholas P Tatonetti, Atul J Butte

**Affiliations:** 1 Department of Pediatrics Bakar Computational Health Sciences Institute, University of California San Francisco, San Francisco, California, USA; 2 Departments of Biomedical Informatics, Systems Biology, and Medicine, Columbia University, New York, New York, USA; 3 Academic Research Systems, Department of Enterprise Data Warehouse University of California San Francisco, San Francisco, California, USA

## Abstract

**Objectives:**

Electronic health record (EHR) data are increasingly used for biomedical discoveries. The nature of the data, however, requires expertise in both data science and EHR structure. The Observational Medical Outcomes Partnership (OMOP) common data model (CDM) standardizes the language and structure of EHR data to promote interoperability of EHR data for research. While the OMOP CDM is valuable and more attuned to research purposes, it still requires extensive domain knowledge to utilize effectively, potentially limiting more widespread adoption of EHR data for research and quality improvement.

**Materials and methods:**

We have created ROMOP: an R package for direct interfacing with EHR data in the OMOP CDM format.

**Results:**

ROMOP streamlines typical EHR-related data processes. Its functions include exploration of data types, extraction and summarization of patient clinical and demographic data, and patient searches using any CDM vocabulary concept.

**Conclusion:**

ROMOP is freely available under the Massachusetts Institute of Technology (MIT) license and can be obtained from GitHub (http://github.com/BenGlicksberg/ROMOP). We detail instructions for setup and use in the Supplementary Materials. Additionally, we provide a public sandbox server containing synthesized clinical data for users to explore OMOP data and ROMOP (http://romop.ucsf.edu).

## BACKGROUND AND SIGNIFICANCE

Widespread adoption of electronic health records (EHRs) into health systems is ushering clinical data into the digital age.^1^ Large-scale analytics of EHR data have produced impactful discoveries that have, in turn, enabled the practice of precision medicine.^2^ It is clear that biomedical researchers in all areas including practicing clinicians, data scientists, and wet lab biologists, could enhance their work by integrating findings taken from this “real world evidence.” There exist many barriers that limit usability of EHR data, however, revolving primarily around available expertise. As EHR data are large scale and typically stored in relational databases, some knowledge of structured query language (SQL) programing is required, which many clinicians and scientists do not have experience with nor the time to sufficiently gain. Furthermore, the structure and data components of EHR systems are complicated and necessitate strong familiarity in order to utilize most effectively. While many get around this issue by setting up effective cross-disciplinary collaborations (eg, physicians working with data science teams), it would be invaluable for enable more researchers themselves to be able work with the data directly.

Further complicating the issue is the fact that different health systems often have different EHR frameworks (eg, Epic or Cerner) and with them, different vocabularies to represent the clinical data. To address issues surrounding interoperability, initiatives like the Observational Health Data Sciences and Informatics (OHDSI; https://www.ohdsi.org/) and Health Level Seven (HL7) have developed common data models (CDMs), such as OHDSI’s Observational Medical Outcomes Partnership (OMOP) CDM or the Fast Healthcare Interoperability Resources (FHIR; https://www.hl7.org/fhir/), to standardize both the language and structure of EHR data. These data models are often built around standardized meta-thesauruses like the Unified Medical Language System (UMLS)^3^ that integrate many different ontologies. One major advantage out of the many to this approach is the ability for researchers across institutions to share code and workflows that will integrate seamlessly.^4^ A substantial amount of research has already leveraged the power of the CDM to perform large-scale observational studies.^5–9^

While the OMOP CDM was built in a framework that is more conducive for research purposes (and is arguably one of the simplest systems of its kind), it still requires a fundamental understanding of EHR data and ontological structures for effective use. Functional and convenient applications, including web tools and software packages that can help visualize data or even perform analyses, can lessen the burden of requiring programing and domain-specific EHR expertise. As of the writing of this manuscript, the OHDSI group has produced an impressive number of packages and resources (over 100 public repositories) on their GitHub page (https://github.com/OHDSI) written in a variety of languages including R, JavaScript, Java, C++, and HTML. These packages cover a range of useful tools beyond just the CDM (eg, CommonDataModel), including back-end engines (eg, ArachneExecutionEngine), connectors (eg, DatabaseConnector), user-interfaces (eg, ArachneUI and AthenaUI), web resources (eg, ATLAS, Achilles, and Athena), tools for data processing and extraction (eg, OhdsiRTools and FeatureExtraction), as well as those that automatically perform analyses (eg, PatientLevelPrection, CohortMethod*,* Cyclops, CaseCrossover, and CaseControl). These packages have been invaluable to the development and dissemination of the OMOP CDM by enabling and encouraging scripts that can automate complex analyses.

These tools effectively work together as a system to enable the entire spectrum of processes starting from OMOP CDM set-up to running experiments. While these tools are immensely useful and powerful, they are not necessarily geared toward new users, which may restrict further adoption of the OMOP CDM. For instance, in order to extract clinical data for a list of patients using FeatureExtraction, a cohort must first be defined using specific outcome IDs in a separate source (eg, ATLAS).

## OBJECTIVES

**Figure 1.**
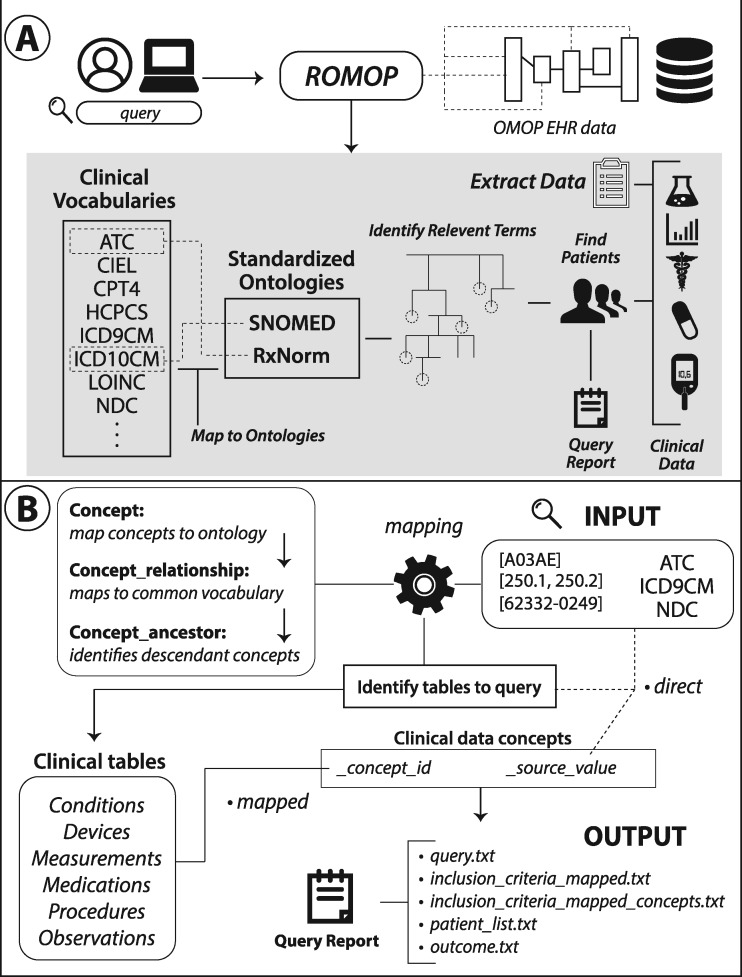
Schematic and structure of ROMOP. (A) ROMOP interfaces with OMOP-formatted EHR data in a relational database structure. Users can retrieve all relevant clinical, demographic, and encounter data for a list of patients directly into an R object as well as search for patients using any vocabulary contained within the OMOP CDM. (B) When searching for patients using clinical vocabularies, ROMOP offers the option to automatically map (“map” option) to the standardized ontology (eg, SNOMED) and query all concepts lower in the hierarchy (ie, descendants). Alternatively users can search for terms directly (“direct” option). ROMOP identifies the relevant clinical tables and fields to query and can produce a set of outputs detailing the query and outcomes. CDM: common data model; EHR: electronic health record; OMOP: Observational Medical Outcomes Partnership.

The goal of ROMOP is to provide a simple and straightforward method to automatically extract relevant clinical and demographic data from an OMOP EHR database into an R object. In this way, ROMOP brings an abstraction layer for EHR data potentially to the millions of R users. Additionally, ROMOP provides a flexible way to search for patients that takes advantage of the power of the unified vocabularies underlying the CDM (ie, finding all patients that are prescribed a pharmaceutical class of medications). In essence, ROMOP combines much of the functionality of current OHDSI packages into a single resource, streamlined for effective EHR research purposes (Figure 1).

In light of interoperability goals, we verified that this package works seamlessly in 2 independent tertiary-care institutions: University of California, San Francisco and Columbia University. This package will ideally supplement other current tools to facilitate more widespread utility of the OMOP data model.

## METHODS

One of the main goals of ROMOP is to facilitate utility of OMOP-formatted EHR data for R users without the need for extensive domain knowledge underlying the CDM. Users of this package need only fundamental expertise of R, general understanding of EHR data (ie, types of vocabularies used to encode data), and minimal exposure to the OMOP CDM. The specific tables from which data can be extracted are relatively straightforward (eg, condition_occurrence for the majority of disease-related data, drug_exposure for medication-related data, etc.). We highly recommend the OHDSI resources and tools mentioned in the Supplementary Materials and sandbox server for users to gain more familiarity with the CDM structure. Further details about the data that are returned and how ROMOP interacts with the CDM can be found in the Supplementary Materials in the *Code Breakdown* section.

### Package details and installation instructions

ROMOP is written in R (version 3.4.1), organized using roxygen2,^10^ and utilizes the following packages for processing: data.table,^11^, DBI,^12^ and dplyr.^13^ ROMOP can connect to an OMOP-formatted EHR database in either MySQL format using odbc^14^ and RMySQL^15^ or a variety of others including, Oracle, PostgreSQL, Microsoft SQL Server, Amazon Redshift, Google BigQuery, and Microsoft Parallel Data Warehouse, made possible through the OHDSI group’s DatabaseConnector^16^ and DatabaseConnectorJars^17^ packages. All queries are automatically translated into the appropriate database syntax using the OHDSI group’s SqlRender^18^ package.

For full details, instructions, and examples please refer to the Readme, which can be obtained from the package GitHub page and is provided in the Supplementary Materials. ROMOP can be retrieved and downloaded from the GitHub page directly or using the following devtools^19^ function: “install_github(“BenGlicksberg/ROMOP”).” In consideration for securely storing connection credential information, these data are entered after installation within the R environment file (“.Renviron”) for retrieval by the package. ROMOP will perform a series of checks to determine whether the server can be connected to and if the required tables are accessible.

### Synthesized clinical EHR data and sandbox server

The Centers for Medicare and Medicaid Services (CMS) have publicly released a synthetic clinical dataset (DE-SynPUF; https://www.cms.gov/Research-Statistics-Data-and-Systems/Downloadable-Public-Use-Files/SynPUFs/DE_Syn_PUF.html) with the aim of being reflective of the patient population, while containing no protected health information (PHI). The OHDSI group previously undertook the task of converting these data into the OMOP CDM format (https://github.com/OHDSI/ETL-CMS). As readers may not have access to EHR data in OMOP format, we provide a sandbox server that has these data in a database where users can explore the CDM as well as the ROMOP package (although users can set up this configuration on their own system following the instructions on the GitHub page). We obtained all data files from ftp://ftp.ohdsi.org/synpuf (accessed June 17, 2018) and created the CDM (DDL and indexes) according to https://github.com/OHDSI/CommonDataModel/tree/master/PostgreSQL, but modified for MySQL. For space considerations, we only uploaded 1 million rows of each of the data files. The sandbox server is a R/shiny^20^ server running as an Elastic Compute Cloud (EC2) instance on Amazon Web Services (AWS) querying a MySQL database server (AWS Aurora MySQL). The interactive tutorial was created using the learnr^21^ package. The sandbox server is available to the public at http://romop.ucsf.edu and contains no PHI.

## RESULTS

While the functionality afforded by this package pales in comparison to the advanced and multifaceted tools provided by the OHDSI group, it will hopefully facilitate EHR data mining for users unfamiliar with the OMOP structure. All package functionality revolves around a data dictionary of all concepts, which prioritizes the tables to query as well as allows for flexible search options (Figure 1A). We provide detailed instructions and example use cases (ie, how to identify all patients prescribed any drug of a certain class) in the package tutorial (see Supplementary Materials) as well interactive examples on the sandbox server.

### Data exploration

ROMOP allows for basic exploration of data types contained in the ontological hierarchy. To understand which vocabularies can be prioritized for queries of interest, the *showDataTypes* function breaks down all available vocabularies per domain (eg, LOINC for Observation and Measurement). The *exploreConcepts* function can be used to identify all mapped terms for a seed concept that are descendants in the hierarchy (see Section 4.3 for more details on what this entails). Additionally, for a provided patient list, ROMOP provides a function to generate a basic demographic breakdown of the cohort.

### Retrieving all data for a set of patients

Researchers who have a preselected patient cohort, or even a single patient of interest, can easily retrieve all relevant clinical, demographic, and encounter information. Relevant demographic (eg, birthdate, gender, and ethnicity) and encounter (eg, visit datetime and type) information can each be retrieved and formatted as an R data.table using a single command. Clinical data are retrieved and mapped to their respective terms from the “condition_occurrence,” “device_exposure,” “drug_exposure,” “measurement,” “procedure_occurrence,” and “observation” tables. All data concepts are first mapped through the data dictionary to return terms instead of identifiers.

### Searching for patient cohort using flexible inclusion (and exclusion) criteria

In the OMOP data structure, there is a distinction between how concepts are recorded and what can be directly searched for. For instance, if the user is interested in the medication idelalisib, it is not possible to directly identify records by searching for the general concept (eg, RxNorm code 1544460) as the data are recorded by the bottom-most (ie, most specific) concepts of the hierarchy (eg, idelalisib 150 MG Delayed Release Oral Tablet). The hierarchical structure of these concepts in the OMOP CDM backend, however, facilitates more powerful searches. In most extracted EHR systems, the user has to define all medications to search, for instance through a prepopulated list or by wildcard string matching (eg, all drug names LIKE “%statin%”). This strategy is ultimately not ideal as it is not extensible to other systems (eg, one system might prescribe a version or formulation of a drug that is not in another) and requires extensive manual quality-control (eg, removing “nystatin” drugs from the string matching results).

As such, we provide straightforward yet flexible ways to search for patients (*findPatients* function) that takes advantage of the underlying OMOP CDM structure (Figure 1B). If the “mapped” option is selected, searching for a broad code like ATC level 3 code A05A (bile therapies), or even a specific term code like RxNorm code 1544460 for idelalisib, will automatically identify and query for all bottom-level (eg, idelalisib 150 MG Delayed Release Oral Tablet) codes contained underneath that seed concept. This works by ROMOP first mapping the initial search criteria to a standard concept (SNOMED or RxNorm) and finding all descendants underneath it through making use of the OMOP concept tables (for more details on this procedure please refer to Supplementary Materials*Code Breakdown* section). Another benefit to this “mapped” option is that terms are not reliant on how the data were originally entered. For instance, if a health system switches from ICD-9CM to ICD-10CM coding, there might be discrepancies in prevalence of codes over time. Mapping to a common concept, however, often alleviates this issue as codes from both vocabularies are typically linked to a common code in the standard vocabulary. The user can also search for the concepts using the “direct” option (ie, search for ICD-9CM code 230.0 only).

This function allows for simultaneous incorporation of multiple vocabulary types (eg, ATC and LOINC codes) and codes, as well as supporting both inclusion and exclusion criteria. The user can also set the strategy of dealing with criteria, namely either union (ie, or) or intersection (ie, and) requirements. Lastly, there is an option to save all output from the search function beyond the patient list, including: the query criteria used; the outcome of the query; and all mapped concepts used for the search along with unique patient counts per term. Please refer to the Readme or Supplementary Materials for more information on the functionality of the package as well as sample more use cases.

## CONCLUSION

In this package, we offer a new straightforward method to interface the popular data science language R with OMOP-formatted EHR data stored in a relational database that is not possible in existing related packages. ROMOP can be used to easily retrieve relevant clinical and demographic data for a patient list of interest. Furthermore, it allows for patient searches using flexible delineations of inclusion and exclusion criteria (ie, multiple and various vocabularies and concept terms) that can take advantage of the power of the CDM.

There are, of course, many limitations to this package. While this package can be run on a server with PHI, these servers are often managed in a restrictive manner and R itself might not always be available. Along these lines, the package will not expand the use of EHR data in and of itself. Users would still be required to have IRB-approved access to EHR data, deidentified or otherwise, structured in the OMOP CDM format in order to use ROMOP. We believe, however, that many research institutions will continue to maintain versions of their EHR data in the OMOP CDM (especially given the relationship between OMOP, the All of Us Research Program, and PCORnet). We are predicting many new data scientists entering the field of clinical data informatics who might appreciate the availability of more straightforward tools for initial exposure to the OMOP CDM. Lastly, we acknowledge the functionality of ROMOP is basic in comparison to the existing powerful tools and packages that can perform complex analyses on OMOP-formatted data. Geared toward new or less experienced users, ROMOP will hopefully supplement many such tools. For individuals without access to OMOP-formatted EHR data, we provide a sandbox server (http://romop.ucsf.edu) to test ROMOP on synthesized clinical EHR data with a step-by-step tutorial. The package and all supporting materials are freely available on GitHub (https://github.com/BenGlicksberg/ROMOP).

## SUPPLEMENTARY MATERIAL

Supplementary material is available at *Journal of the American Medical Informatics Association* online.

## CONTRIBUTORS

B.S.G. designed the study, authored the software package and sandbox system tutorial, and wrote the manuscript. B.O. provided system administration infrastructural support and guidance for implementing the package on UCSF’s EHR as well as setting up and configuring the public sandbox server. V.R. and D.D. provided clinical expertise in design and utilization of the package as well as framing the manuscript. R.F., N.L., and R.L. were responsible for enabling utility of UCSF EHR data in OMOP format. N.G., P.T., and N.P.T. deployed and tested the package using Columbia University’s EHR and provided software and manuscript edits and revisions. A.J.B. conceived of the study, provided overall guidance and support, and made critical manuscript revisions. All authors reviewed and approved of the manuscript.

## Supplementary Material

Supplementary Data
