# Supplementary material for: ROMOP: a light-weight R package for interfacing with OMOP-formatted electronic health record data
Source: JAMIA Open. 2019 Jan 4;2(1):10–4. doi: 10.1093/jamiaopen/ooy059 (PMC6800657; doi:10.1093/jamiaopen/ooy059)
Supplement: Supplementary Data [file ooy059_supp.pdf]

ROMOP Readme

Benjamin S. Glicksberg

9/14/2018

Contents

ROMOP . . . 2
Sandbox Server . . . 2
Requirements . . . 3
Clinical Data . . . 3
Programming Language . . . 3
Installation . . . 3
Download . . . 3
Setup . . . 4
Credentials . . . 4
Checks . . . 4
On start . . . 4
Functions . . . 5
Utility . . . 5
getDemographics . . . 5
getEncounters . . . 5
getClinicalData . . . 6
findPatients . . . 6
Misc. . . . 8
changeOutDirectory . . . 8
makeDataOntology . . . 8
summarizeDemographics . . . 9
showDataTypes . . . 9
exploreConcepts . . . 9
Examples . . . 10
Simple . . . 10
Advanced . . . 11
Output . . . 11
query.txt . . . 12
inclusion_criteria_mapped.txt . . . 12
inclusion_criteria_mapped_concepts.txt . . . 12
exclusion_criteria_mapped.txt . . . 12
exclusion_criteria_mapped_concepts.txt . . . 13
outcome.txt . . . 13
patient_list.txt . . . 13
Code Breakdown . . . 13
Data Retrieval . . . 13
Searching . . . 13
Helpful Hints . . . 15
License . . . 15
Contact . . . 16

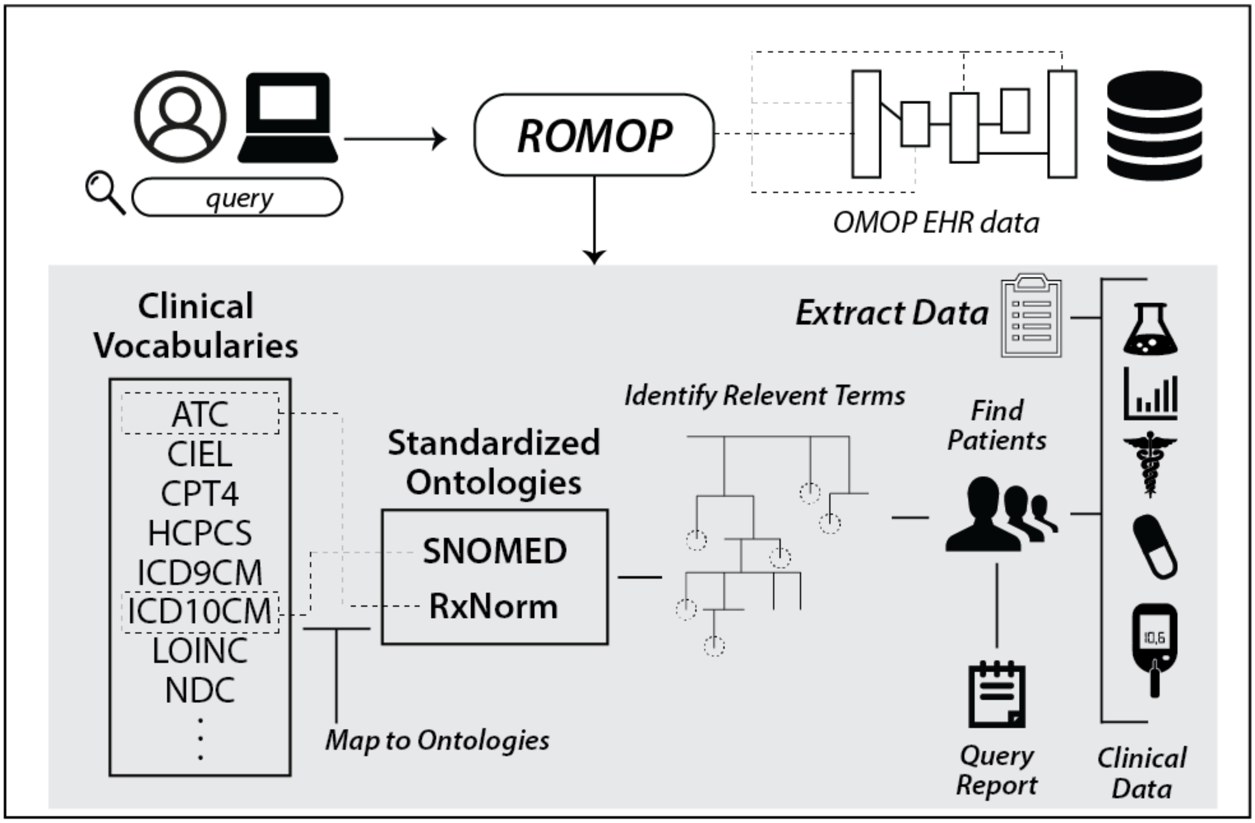

Figure 1: Features of ROMOP

ROMOP

ROMOP is a flexible R package to interface with the Observational Health Data Sciences and Informatics (OHDSI) OMOP Common Data Model. Briefly, OMOP is a standardized relational database schema for Electronic Health Record (EHR) or Electronic Medical Record (EMR) data (i.e., patient data collected during clinical visits to a health system). The main benefit of a standardized schema is that it allows for interoperability between institutions, even if the underlying EHR vendors are disparate.

For a detailed description of the OMOP common data model, please visit this helpful wiki.

In its backend, OMOP relies on standardized data ontologies and metathesaureses, such as the Unified Medical Language System (UMLS), and as such, the queries within ROMOP heavily rely on these vocabularies. Athena is a great tool to better understand the concepts in these ontologies and identify ideal search terms of interest.

Manuscript information:
Glicksberg BS, Oskotsky B, Giangreco Nˆ, Thangaraj PMˆ, Rudrapatna V, Datta D, Frazier R, Lee N, Larsen R, Tatonetti NP, Butte1 AJ: ROMOP: a light-weight R package for interfacing with OMOP-formatted Electronic Health Record data (in review)

Sandbox Server

The Centers for Medicare and Medicaid Services (CMS) have released a synthetic clinical dataset DE-SynPUF) in the public domain with the aim of being reflective of the patient population but containing no protected health information. The OHDSI group has underwent the task of converting these data into the OMOP CDM

format. Users are certainly able to set up this configuration on their own system following the instructions on the GitHub page. We obtained all data files from the OHDSI FTP server (accessed June 17th, 2018) and created the CDM (DDL and indexes) according to their official instructions, but modified for MySQL. For space considerations, we only uploaded one million rows of each of the data files. The sandbox server is a Rshiny server running as an Elastic Compute Cloud (EC2) instance on Amazon Web Services (AWS) querying a MySQL database server (AWS Aurora MySQL).

Requirements

Clinical Data

ROMOP requires EHR data to be in OMOP format and on a server accessible to by the user. In it's current form, ROMOP can connect to databases in *MySQL* using the RMySQL driver or many other formats, including *Oracle*, *PostgreSQL*, *Microsoft SQL Server*, *Amazon Redshift*, *Google BigQuery*, and *Microsoft Parallel Data Warehouse*, through utilization of the DatabaseConnector and SqlRender packages developed by the OHDSI group (see below).

Users without access to EHR data might consider using synthetic public data following the instructions provided by the OHDSI group here.

Programming Language

ROMOP is built in the R environment and developed on version 3.4.4 (2018-03-15).

ROMOP requires the following R packages:

- DBI (developed on version 1.0.0)
- data.table (developed on version 1.10.4-3).
- dplyr (developed on version 0.7.4).

Driver-specific:

- RMySQL (developed on version 0.10.14).
- DatabaseConnector (developed on version 2.2.0)
- DatabaseConnectorJars (developed on version 1.0.0)
- SqlRender (developed on version 1.5.2)

Installation

Download

ROMOP can be installed easily from github using the devtools package:

```
library(devtools)
install_github("BenGlicksberg/ROMOP")
```

Alternatively, the package can be downloaded directly from the github page and installed by the following steps:

1. Unzip ROMOP-master.zip
2. R CMD INSTALL ROMOP-master

Please see the Setup section to properly configure the package to work.

Setup

Credentials

In accordance with best practices for storing sensitive information, credentials are not saved in plain text but in the .Renviron file. A formatted .Renviron file is provided with the package with the following fields to fill in:

```
driver = ""
host = ""
username = ""
password = ""
dbname = ""
port = "3306"
```

- driver (case insensitive): "mysql" for MySQL or (according to OHDSI DatabaseConnector package) "postgresql" for PostgreSQL, "oracle" for Oracle, "sql server" for Microsoft SQL Server, "redshift" for Amazon Redshift, "pdw" for Microsoft Parallel Data Warehouse, or "bigquery" for Google BigQuery.

- host (or server depending on database format)

- dbname: OMOP EHR database name (or schema depending on database format)

Note that this .Renviron file has to be in the same directory where R is launched. If already using an .Renviron file, add this information to it.

Checks

With credentials correctly configured, the package can be loaded. ROMOP will now check for 3 conditions to be met:

1. Check that the credentials exist and can be retrieved from .Renviron file:
   requires driver, host, username, password, dbname, and port exist
2. Check that connection to OMOP EHR server and database can be made:
   uses the above credentails
3. Check to ensure all required OMOP tables exist and contain (any) data:
   the required tables are:

```
"concept","concept_ancestor","concept_relationship","condition_occurrence","death",
"device_exposure","drug_exposure","measurement","observation","person","procedure_occurrence","visit_oc
```

- if any of the above tables are missing, a warning message will be produced and the package will not be able to load properly.
- if any of the above tables exist, but do not contain any data, a warning message will be produced but the package will still be able to function.

On start

Successfully pasing all checks will allow the user to begin using ROMOP.

1. Set an output directory to use with the changeOutDirectory function (note: the default output directory will be declared on package load).

2. Create/load the Data ontology (required to decode data types) using the makeDataOntology. For the first time running this package, the concept ontology will have to first be built, but if the store_ontology option is selected, the ontology will be saved as an .rds file for subsequent loading.

Functions

Utility

getDemographics

Description: Retrieves and formats patient demographic data from the **person** and **death** tables. Option to restrict to patientlist of interest.

Usage: ptDemo <- getDemographics(patient_list=NULL,declare=TRUE)

Arguments:

patient_list *comma-separated string of patient ids*
a provdied patientlist will restrict search to ids. NULL will return demographic data for all available patients

declare *TRUE/FALSE*
if TRUE, outputs status and updates to the screen

Value:

Returns a data.table with demographic data: person_id, birth_datetime, age, Gender, Race, Ethnicity, death_date, Status (Alive/Deceased)

Details:

- patient_list should be in the following format: "patient_id_1, patient_id_2, . . . "

getEncounters

Description: Retrieves and formats patient encounter data from the **visit_occurrence** table. Requires patientlist input.

Usage: ptEncs <- getEncounters(patient_list,declare=TRUE)

Arguments:

patient_list *comma-separated string of patient ids*
searches for all encounter data for the patientlist inout.

declare *TRUE/FALSE*
if TRUE, outputs status and updates to the screen

Value:

Returns a data.table with encounter data: person_id, visit_occurrence_id, visit_start_datetime, visit_end_datetime, visit_source_value, visit_concept, visit_source_concept, admitting_concept, discharge_concept

Details:

- patient_list should be in the following format: "patient_id_1, patient_id_2, . . . "

getClinicalData

Description: Retrieves all relevant clinical data for individuals in a patientlist. Wrapper for domain-specific getData functions (which can also be used separately).

Usage: ptClinicalData <- getClinicalData(patient_list, declare=TRUE)

Arguments:

patient_list *comma-separated string of patient ids*
a provdied patientlist will restrict search to ids. NULL will return demographic data for all available patients

declare *TRUE/FALSE*
if TRUE, outputs status and updates to the screen

Value:
Returns a list of data.tables stratified by domain type (e.g., ptClinicalData$Condition, ptClinicalData$Observation, etc. . .)

Details:

- patient_list should be in the following format: "patient_id_1, patient_id_2, . . . "
- getClinicalData calls domain-specific getData functions for the following domains: Observation, Condition, Procedure, Medication (Drug), Measurement, and Device. Each function can also be run individually (e.g, getConditions; getMedications).
- In addition to datetimes, visit_occurrence_ids, _concept_ids and _source_concept_ids, other domain-specific concepts and values are retrieved and mapped:
  - Observation: observation_type_concept, value_as_number, value_as_string, value_as_concept, unit_source_value
  - Condition: condition_type_concept, condition_status
  - Procedure: procedure_type_concept, quantity,
  - Medication: drug_type_concept, stop_reason, refills, quantity, days_supply, sig, route_concept, effective_drug_dose, dose_unit_concept, route_source_value, frequency, frequency_unit, rx_quantity_unit_source_value
  - Measurement: measurement_type_concept, value_as_number, value_as_concept, unit_concept
  - Device: device_type_concept

findPatients

Description: Main function to identify patients based on clinical data inclusion (and exclusion, if desired) criteria. Flexible to allow for multiple data types, vocabularies, and concepts.

Usage: patientlist <- findPatients(strategy_in="mapped", vocabulary_in, codes_in, function_in = "or", strategy_out = NULL, vocabulary_out = NULL, codes_out = NULL, function_out = NULL, declare=FALSE, save=FALSE, out_name=NULL)

Arguments:

strategy_in *mapped* or *direct*
dictates the strategy for how inclusion criteria are treated (see Details).

vocabulary_in *vocabularies for inclusion criteria*
comma-separated string of relevant vocabularies for inclusion criteria (see Details).

codes_in *specific concept codes for inclusion criteria*
semi-colon separated string of code concepts for inclusion criteria, corresponding to the order for vocabulary_in. Multiple codes can be used per vocabulary and should be comma-separated (see Details).

function_in *and* or *or*
dictates how multiple inclusion should be treated. *and* necessitates that all inclusion criteria are met (i.e., intersection), while *or* allows for any critera to be met (i.e., union) (see Details).

strategy_out *mapped* or *direct* or NULL (default)
dictates the strategy for how exclusion are treated. NULL indicates no exclusion criteria.

vocabulary_out *vocabularies for exclusion criteria* or NULL (default)
comma-separated string of relevant vocabularies for exclusion criteria. NULL indicates no exclusion criteria.

codes_out *specific concept codes for exclusion criteria* or NULL (default)
semi-colon separated string of code concepts for inclusion criteria, corresponding to the order for vocabulary_out. Multiple codes can be used per vocabulary and should be comma-separated. NULL indicates no exclusion criteria.

function_out *and* or *or* or NULL
dictates how multiple exclusion should be treated. *and* necessitates that all exclusion criteria are met (i.e., intersection), while *or* allows for any critera to be met (i.e., union). NULL indicates no exclusion criteria.

declare *TRUE/FALSE*
if TRUE, outputs status and updates to the screen.

save *TRUE/FALSE*
if TRUE, various query output saved to outDirectory (see Details).

out_name *name assigned to search query* or NULL
if save == TRUE, saves query using provided name. If the provided name already exists as a directory (or is NULL), the directory defaults to datetime name (see Details).

Value:
Returns a list of patients that meet inclusion criteria (and not exclusion criteria if entered).

Details:

- *direct* strategy queries the concepts directly by _source_concept in clinical tables. *mapped* maps to common ontology (via **concept_synonym**) and identifies relevant descendants (via **concept_ancestor**) to search for in _concept fields.
- the exploreConcepts function can be used to find ideal concepts to search for.

- vocabulary_ input for multiple inputs should use relevant vocabularies (see showDataTypes) as a comma-separated string, e.g., "ATC, ICD10CM, SNOMED".
- codes_ input correspond to the order as the vocabulary_ input and should be semi-comma separated string in the same order as above. Multiple terms per vocabulary type should be comma-separated. e.g., "A01A; K50, K51; 235599003" correspond to "A01A" for ATC, "K50" and "K51" for ICD10CM, and "235599003" for SNOMED.

- function_ corresponds to how criteria should be treated. *and* necessitates patients meet all criteria while *or* allows for patients to meet any of the criteria.
- Please note that if no standard common concepts are found per search domain, a warning message will appear and the search will not be able to be performed (see Helpful Hints for more details.)
- if save == TRUE, the following information is saved in a directory per query:

– query: all arguments for the search.
– _criteria_mapped: all original criteria for inclusion (and exclusion if applicable) that are mapped to dataOntology.
– criteria_mapped_concepts: all mapped concepts used for inclusion (and exclusion if applicable) that are used to search in clinical data tables. Additionally, the pt_count column displays the number of unique patients that have a record with the corresponding concept.

– outcome: results of the search (most relevant when exclusion criteria are applied).

– patient_list: list of patients that meet inclusion (and not exclusion, if applicable) criteria.

Misc.

changeOutDirectory

Description: Sets the current outDirectory which will store the Data Ontology and all function output. Option to create directory if does not exist.

Usage: changeOutDirectory(outdir="path/to/directory", create=FALSE)

Arguments:
outdir directory path

create *TRUE/FALSE*
will create the directory if it does not exist

Value:
Nothing returned; simply sets (and creates if set to) output directory

Details:

- If directory does not exist and create=FALSE, a warning message will appear and the output directory will not be changed.

makeDataOntology

Description: Creates general Data Ontology used by all data tables from the **concept** table. Option to save/load.

Usage: dataOntology <- makeDataOntology(declare=TRUE,store_ontology=FALSE)

Arguments:
declare *TRUE/FALSE*
if TRUE, outputs status and updates to the screen

store_ontology *TRUE/FALSE*
if TRUE, will save/load the ontology instead of active querying

Value:
Returns a data.table with concept data.

Details:

- Generating the Data Ontology takes ~31.2 secs and is ~491.6 Mb.

- If declare == TRUE, the following information will be returned:

```
Retrieving concept data...
Concept data loaded; data found for:
## unique domains.
## unique vocabularies.
### unique concept classes.
```

- If store_ontology == TRUE, attempts to load from memory (in the outDirectory) and saves if does not exist (~53 Mb). Loading takes ~8 secs.

summarizeDemographics

Description: Summarizes patient demographic data from the getDemographics function.

Usage: summarizeDemographics(ptDemo)

Arguments:

ptDemo *patient demographics table*
ptDemo is the patient demographics object from the getDemographics function output

Value:

N/A; outputs message with descriptive summary statistics for the relevant patient demographic data.

showDataTypes

Description: Details relevant vocabularies per domain. Requires dataOntology to have been created (via makeDataOntology).

Usage: showDataTypes()

Arguments:

N/A

Value:

Returns a table of vocabularies contained within clinical domains: Condition, Observation, Measurement, Device, Procedure, Drug.

exploreConcepts

Description: For given vocabulary and concept, returns the mapped standard concept(s) as well as decendent concept(s)

Usage: conceptsInfo <- exploreConcepts(vocabulary, codes)

Arguments:

vocabulary *vocabulary*
comma-separated string of relevant vocabularies for inclusion criteria (see Details).

codes *concept codes*
semi-colon separated string of code concepts for inclusion criteria, corresponding to the order for vocabulary. Multiple codes can be used per vocabulary and should be comma-separated (see Details).

Value:

Returns a table of concepts contained under (i.e., below in the heirarchy) the query concept.

Details:

- vocabulary input for multiple inputs should use relevant vocabularies (see showDataTypes) as a comma-separated string, e.g., "ATC, ICD10CM".
- codes input correspond to the order as the vocabulary input and should be semi-comma separated string in the same order as above. Multiple terms per vocabulary type should be comma-separated. e.g., "A01A; K50, K51" correspond to "A01A" for ATC and "K50" and "K51" for ICD10CM.

Examples

Both simple and advanced findPatients queries will be outlined. See the Output section for description of output if save == TRUE. For the process timing provided, all queries were run on an Amazon Elastic Compute Cloud (EC2) instance.

Simple

1. Disease category (ICD10CM): find all "Type 2 Diabetes Mellitus" patients (E11)

Here we will set a single inclusion criterion. The inclusion vocbulary is set to *ICD10CM* and the inclusion code is *E11* corresponding to the vocabulary. Because the inclusion strategy is set as "mapped", ROMOP will map the ICD10CM code to a common ontology (SNOMED) term and find all descendants to search for (see Code Breakdown for details on how this works).

query

```
patient_list = findPatients(strategy_in="mapped", vocabulary_in = "ICD10CM", codes_in = "E11")
```

time: 15.3 secs

2. Specific disease (ICD9CM): find all patients with "Diabetes with ketoacidosis, type I [juvenile type], not stated as uncontrolled" **only** (250.11)

Here we will search for patients that have the specific *ICD9CM* code *250.11* **only**, i.e., not map to common ontology (see Code Breakdown for the importance of this distiction).

query

```
patient_list = findPatients(strategy_in="direct", vocabulary_in = "ICD9CM", codes_in = "250.11")
```

time: 1.1 min

3. Multiple diseases (ICD10CM): find all patients with "Essential (primary) hypertension" (I10) **and** "Angina pectoris with documented spasm" (I20.1)

Here we will search for patients that have the multiple ICD10CM codes. While we put a single inclusion vocabulary, we will put two inclusion codes separated by a comma. Also we set the inclusion function to "and" which requires **both** criteria to be met.

query

```
patient_list = findPatients(strategy_in="mapped", vocabulary_in = "ICD10CM", codes_in = "I10, I20.1", f
```

time: 23.8 secs

4. Drug class (ATC): find all patients prescribed with any "Serotonin receptor antagonists" (A03AE)

Here we will search for patients by drug ATC code. As the inclusion strategy is set to "mapped", all drugs that fall into this category will automatically be identified and searched for (see Code Breakdown for details on how this works).

query

```
patient_list = findPatients(strategy_in="mapped", vocabulary_in = "ATC", codes_in = "A03AE")
```

time: 1.1 secs

5. Disease category (ICD10CM) but not Drug (MeSH): find all patients with "Other anxiety disorders" (F31), but *not* prescribed with "Clonazepam" (D002998)

Here we will search for patients by ICD10CM code as before. We also identify all patients prescribed with the MeSH term for "Clonazepam", which will be removed from the original list.

query

```
patient_list = findPatients(strategy_in="mapped", vocabulary_in = "ICD10CM", codes_in = "F41", strategy_
```

time: 16.5 secs

Advanced

1. Multiple disease categories (ICD10CM) and lab test (LOINC) but not multiple disease categories (ICD10CM) nor drug class (RxNorm): find all patients with "Crohn's disease" (F31) and "Malignant neoplasm of prostate" (C61) with "CBC W Auto Differential panel - Blood" (57021-8), but *not* "Gastroenteritis and colitis due to radiation" (K52.0) nor "Allergic and dietetic gastroenteritis and colitis" (K52.2) nor prescribed with any "Aminosalicylate" (113374)

Here we will search for patients by ICD10CM code as before. We also identify all patients prescribed with the MeSH term for "Clonazepam", which will be removed from the original list.

query

```
vocabulary_in = "ICD10CM, LOINC"
codes_in = "K50;C61, 57021-8"
vocabulary_out = "ICD10CM, RxNorm"
codes_out = "K52.0; K52.2,  113374"

patient_list = findPatients(strategy_in="mapped", vocabulary_in = vocabulary_in, codes_in = codes_in, f
```

time: 5.9 mins

Output

All output is saved in the output directory (use changeOutDirectory to set). Additionally, the data ontology file will be loaded from here and saved if set to using the makeDataOntology](#makedataontology) function.

If save==TRUE is selected for findPatients queries, various information will be saved in a created query-specific directory within the outDirectory:
+ query: all arguments for the search. + _criteria_mapped: all original criteria for inclusion (and exclusion if applicable) that are mapped to dataOntology. + criteria_mapped_concepts: all mapped concepts used for inclusion (and exclusion if applicable) that are used to search in clinical data tables. Additionally, the pt_count column displays the number of unique patients that have a record with the corresponding concept.
+ outcome: results of the search (most relevant when exclusion criteria are applied).
+ patient_list: list of patients that meet inclusion (and not exclusion, if applicable) criteria.

We will detail the respective output files that are derived from Simple Examples #5:

query.txt

```
cat query.txt
```

```
inclusion strategy: mapped
inclusion vocabularies: ICD10CM
inclusion codes: F41
inclusion function: or
exclusion strategy: mapped
exclusion vocabularies: MeSH
exclusion codes: D002998
exclusion function: and
```

inclusion_criteria_mapped.txt

```
cat inclusion_criteria_mapped.txt
```

```
codes   vocabularies    concept_id  concept_name    domain_id   vocabulary_id   concept_class_id
F41 ICD10CM 1568230 Other anxiety disorders Condition   ICD10CM 3-char nonbill code
```

inclusion_criteria_mapped_concepts.txt

```
head inclusion_criteria_mapped_concepts.txt
```

```
descendant_concept_id   ancestor_concept_id concept_name    domain_id   vocabulary_id   concept_class_i
381537  442077  Organic anxiety disorder    Condition   SNOMED  Clinical Finding    17496003    NA
432600  442077  Stress reaction causing mixed disturbance of emotion and conduct    Condition   SNOMED
433178  442077  Anxiety disorder of childhood OR adolescence    Condition   SNOMED  Clinical Finding
434613  442077  Generalized anxiety disorder    Condition   SNOMED  Clinical Finding    21897009    NA
434628  442077  Separation anxiety  Condition   SNOMED  Clinical Finding    126943008   NA
436074  442077  Panic disorder  Condition   SNOMED  Clinical Finding    371631005   NA
436390  442077  Psychogenic rumination  Condition   SNOMED  Clinical Finding    192014006   NA
436676  442077  Posttraumatic stress disorder   Condition   SNOMED  Clinical Finding    47505003    NA
437537  442077  Shyness disorder of childhood   Condition   SNOMED  Clinical Finding    83253003    NA
```

exclusion_criteria_mapped.txt

```
cat exclusion_criteria_mapped.txt
```

```
codes   vocabularies    concept_id  concept_name    domain_id   vocabulary_id   concept_class_id
D002998 MeSH    45612901    Clonazepam  Drug    MeSH    Main Heading
```

exclusion_criteria_mapped_concepts.txt

```
head exclusion_criteria_mapped_concepts.txt
```

```
descendant_concept_id   ancestor_concept_id concept_name    domain_id   vocabulary_id   concept_class_i
798874  798874  Clonazepam  Drug    RxNorm  Ingredient  2598    NA
798875  798874  Clonazepam 0.5 MG Oral Tablet   Drug    RxNorm  Clinical Drug   197527  NA
798876  798874  Clonazepam 1 MG Oral Tablet Drug    RxNorm  Clinical Drug   197528  NA
798877  798874  Clonazepam 2 MG Oral Tablet Drug    RxNorm  Clinical Drug   197529  NA
798893  798874  Clonazepam 0.125 MG Oral Tablet [Klonopin]  Drug    RxNorm  Branded Drug    211761  NA
798894  798874  Clonazepam 0.25 MG Oral Tablet [Klonopin]   Drug    RxNorm  Branded Drug    211762  NA
798896  798874  Clonazepam 1 MG/ML Injectable Solution  Drug    RxNorm  Clinical Drug   249943  NA
798897  798874  Clonazepam 0.5 MG   Drug    RxNorm  Clinical Drug Comp  315699  NA
798899  798874  Clonazepam 2 MG Drug    RxNorm  Clinical Drug Comp  317336  NA
```

outcome.txt

```
cat outcome.txt
```

```
# patients found from the inclusion criteria ONLY.
# patients found from the exclusion criteria ONLY.
# overlapping patients excluded from the original inclusion input based on the exclusion criteria.
# patients found that meet the inclusion and exclusion criteria.
```

patient_list.txt

```
head patient_list.txt
```

```
patient_list
1
2
3
```

Code Breakdown

ROMOP first requires the creation a data dictionary (using makeDataOntology function) of the ontology (from *concept* table) that is referenced and utilized to map to all concepts for all functions. Using this ontology, all searches and extractions are optimized to only query tables in which the data could be found.

Data Retrieval

The majority of data in clinical tables are stored as concepts. When data is extracted, ROMOP first maps the relevant concepts (e.g., device_type_concept_id) to the data dictionary and then returns the mapped concepts to the user.

Searching

In the OMOP data structure, there is a distinction between how concepts are recorded and what can be directly searched for. For instance, if the user is interested in the medication idelalisib, it is not possible to directly identify records by searching for the general concept (e.g., RxNorm code 1544460) as the data are recorded by the bottom-most (i.e., most specific) concepts of the hierarchy (e.g., idelalisib 150 MG Delayed Release Oral Tablet). The hierarchical structure of these concepts in the OMOP CDM back-end,

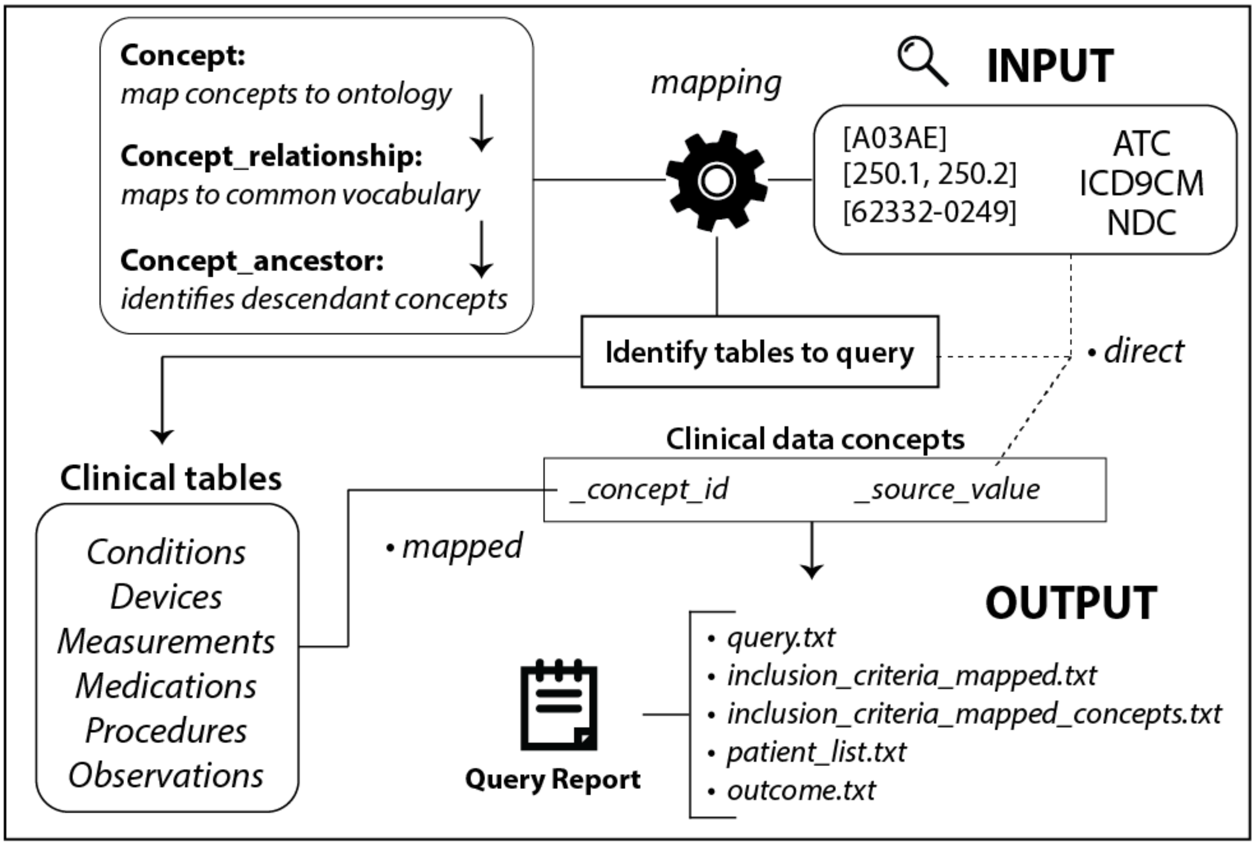

Figure 2: Workflow of ROMOP functionality

however, facilitates more powerful searches. In most extracted EHR systems, the user has to define all medications to search, for instance through a pre-populated list or by wildcard string matching (e.g., all drug names LIKE "%statin%"). This strategy is ultimately not ideal as it is not extensible to other systems (e.g., one system might prescribe a version or formulation of a drug that is in not in another) and requires extensive manual quality-control (e.g., removing "nystatin" drugs from the string matching results). For the findPatients function, if the "mapped" option is selected, searching for a broad code like ATC level 3 code A05A (bile therapies), or even a specific term code like RxNorm code 1544460 for idelalisib, will automatically identify and query for all bottom-level (e.g., idelalisib 150 MG Delayed Release Oral Tablet) codes contained underneath that seed concept. This works by ROMOP first mapping the initial search criteria to a standard concept (SNOMED or RxNorm) and finding all descendants underneath it. Another benefit to this "mapped" option is that terms are not reliant on how the data were originally entered. For instance, if a health system switches from ICD-9CM to ICD-10CM coding, there might be discrepancies in prevalence of codes over time. Mapping to a common concept, however, often alleviates this issue as codes from both vocabularies are typically linked to a common code in the standard vocabulary. Of course the user can search for the concepts they entered only using the "direct" option (i.e., search for ICD-9CM code 230.0 only).

Helpful Hints

- We recommend using the *mapped* argument for the findPatients function because the concepts will not depend on by which format the data was entered (i.e., the *source_concept*). This is important as diffierent institutions may utilize different underlying terminologies, as well as switch primary data entry vocabularies over time (i.e., the switch from ICD-9 to ICD-10). For example, if the user is interested in "Trigeminal neuralgia", using the ICD-10 code "G50.1" with the *direct* argument, all prior entries that utilized the corresponding ICD-9 code ("350.1") most likely will not be found as many data warehouses do not "back-map" codes. Using the *mapped* argument will bypass this issue as the standard concept will be used which should capture both options.
- Standard vocabularies: while the OMOP common data model utilizes many ontologies, **SNOMED** and **RxNorm** are used primarily for common concepts in the clincal data tables. As such, while any vocabulary can be used for findPatients, the *mapped* function will only be able to find data contained within the following common concepts per domain:

```
##   domain_type                  concepts
## 1 Measurement       LOINC,SNOMED,CPT4
## 2   Condition                  SNOMED
## 3        Drug           RxNorm,CPT4,NDC
## 4 Observation SNOMED,CPT4,LOINC,HCPCS
## 5      Device             SNOMED,HCPCS
## 6   Procedure       SNOMED,CPT4,HCPCS
```

Consequently, if inclusion/exclusion criteria can be be mapped to the data ontology, but no synonym/descendants are contained within the above common concepts, no search will be performed (as no patients would be returned). This most directly affects searching for *Drug* concepts, in which we reccommend not using standard common concepts (e.g., RxNorm, ATC) for search criteria.

- To ensure complete capture of data concepts of interest, we recommend identifying multiple vocabulary/codes to use using the Athena resource. For instance, if interested in finding all individuals taking a Benzodiazepine, consider using both the relevant ATC classes (e.g., N03AE) as well as the relevant Substance (SNOMED) codes (e.g., 16047007). The exploreConcepts function can be used to identify and prioiritize which codes are optimal to use.

License

MIT License

Copyright (c) 2018 Benjamin S. Glicksberg

Permission is hereby granted, free of charge, to any person obtaining a copy of this software and associated documentation files (the “Software”), to deal in the Software without restriction, including without limitation the rights to use, copy, modify, merge, publish, distribute, sublicense, and/or sell copies of the Software, and to permit persons to whom the Software is furnished to do so, subject to the following conditions:

The above copyright notice and this permission notice shall be included in all copies or substantial portions of the Software.

THE SOFTWARE IS PROVIDED “AS IS”, WITHOUT WARRANTY OF ANY KIND, EXPRESS OR IMPLIED, INCLUDING BUT NOT LIMITED TO THE WARRANTIES OF MERCHANTABILITY, FITNESS FOR A PARTICULAR PURPOSE AND NONINFRINGEMENT. IN NO EVENT SHALL THE AUTHORS OR COPYRIGHT HOLDERS BE LIABLE FOR ANY CLAIM, DAMAGES OR OTHER LIABILITY, WHETHER IN AN ACTION OF CONTRACT, TORT OR OTHERWISE, ARISING FROM, OUT OF OR IN CONNECTION WITH THE SOFTWARE OR THE USE OR OTHER DEALINGS IN THE SOFTWARE.

Contact

For questions, comments, errors, bug reports, or issues, please contact: benjamin.glicksberg@ucsf.edu
For general correspondance, please contact: atul.butte@ucsf.edu
